# The influence of surface chemistry on the kinetics and thermodynamics of bacterial adhesion

**DOI:** 10.1038/s41598-018-35343-1

**Published:** 2018-11-22

**Authors:** Jun Kyun Oh, Yagmur Yegin, Fan Yang, Ming Zhang, Jingyu Li, Shifeng Huang, Stanislav V. Verkhoturov, Emile A. Schweikert, Keila Perez-Lewis, Ethan A. Scholar, T. Matthew Taylor, Alejandro Castillo, Luis Cisneros-Zevallos, Younjin Min, Mustafa Akbulut

**Affiliations:** 1 Artie McFerrin Department of Chemical Engineering, Texas A&M University, College Station, Texas 77843 USA; 2 Department of Nutrition and Food Science, Texas A&M University, College Station, Texas 77843 USA; 3 Department of Chemistry, Texas A&M University, College Station, Texas 77843 USA; 4 Department of Polymer Engineering, University of Akron, Akron, Ohio 44325 USA; 5 Department of Animal Science, Texas A&M University, College Station, Texas 77843 USA; 6 Department of Horticultural Sciences, Texas A&M University, College Station, Texas 77843 USA; 7 Department of Materials Science and Engineering, Texas A&M University, College Station, Texas 77843 USA

## Abstract

This work is concerned with investigating the effect of substrate hydrophobicity and zeta potential on the dynamics and kinetics of the initial stages of bacterial adhesion. For this purpose, bacterial pathogens *Staphylococcus aureus* and *Escherichia coli* O157:H7 were inoculated on the substrates coated with thin thiol layers (i.e., 1-octanethiol, 1-decanethiol, 1-octadecanethiol, 16-mercaptohexadecanoic acid, and 2-aminoethanethiol hydrochloride) with varying hydrophobicity and surface potential. The time-resolved adhesion data revealed a transformation from an exponential dependence to a square root dependence on time upon changing the substrate from hydrophobic or hydrophilic with a negative zeta potential value to hydrophilic with a negative zeta potential for both pathogens. The dewetting of extracellular polymeric substances (EPS) produced by *E*. *coli* O157:H7 was more noticeable on hydrophobic substrates, compared to that of *S*. *aureus*, which is attributed to the more amphiphilic nature of staphylococcal EPS. The interplay between the timescale of EPS dewetting and the inverse of the adhesion rate constant modulated the distribution of *E*. *coli* O157:H7 within microcolonies and the resultant microcolonial morphology on hydrophobic substrates. Observed trends in the formation of bacterial monolayers rather than multilayers and microcolonies rather than isolated and evenly spaced bacterial cells could be explained by a colloidal model considering van der Waals and electrostatic double-layer interactions only after introducing the contribution of elastic energy due to adhesion-induced deformations at intercellular and substrate-cell interfaces. The gained knowledge is significant in the context of identifying surfaces with greater risk of bacterial contamination and guiding the development of novel surfaces and coatings with superior bacterial antifouling characteristics.

## Introduction

Bacterial fouling causes not only the transmission of infection and disease from one surface to another and humans, but also the reduction in the operational function, sustainability, and efficiency of various types of surfaces and devices. For instance, hospital-acquired infections arise, or may be predicated upon, from the cross-contamination of surgical tools, medical implants, and surfaces within the healthcare environment (i.e., surfaces within and adjacent to patient care areas) with bacterial pathogens^1–3^. According to the World Health Organization, human foodborne illnesses caused by *Salmonella enterica* serovars, *Campylobacter* species, *Clostridium perfringens*, *Listeria monocytogenes*, *Escherichia coli* O157, *Shigella* species, and *Vibrio cholerae* account for a median of about 350 million illnesses and about 190,000 deaths per annum^4^. The use of contact lenses can result in bacterial contamination and the development of microbial keratitis^5^. Fouling of reverse osmosis membranes by marine bacteria is a major problem in seawater desalination^6,7^. Maritime vehicles can experience an increased hydrodynamic drag and friction coefficient owing to bacterial attachment and growth and the resulting perturbations of flow fields around the submerged surfaces of these vehicles^8,9^. The contact of sulfate-reducing bacteria with metallic surfaces under anoxic conditions can lead to microbially influenced corrosion, which has widely recognized adverse technical and economic effects^10^. Bacterial contamination in the food industry, in particular, during processing of fruits and vegetables concerning the increased resistance of bacteria to chemicals used in cleaning, disinfection, and sanitization^11–13^.

Because bacterial adhesion to the surface is an initial prerequisite to bacterial fouling, understanding how surface characteristics influence adhesion processes is crucial to identify surfaces with greater ability to support bacterial adhesion, and also to develop novel surfaces and coatings with bacterial antifouling characteristics^14–16^. The current consensus is that the substrate (which refers to abiotic surface throughout this manuscript) hydrophobicity and zeta potential are important physicochemical parameters controlling the bacterial adhesion on them^17–20^. The adhesive forces between substrate and bacterium arise through van der Waals and electrostatic double-layer interactions^21–23^, while the additional consideration of acid-base interactions has been reported to better correlate the experimental observations and thermodynamic predictive models^24,25^. Several studies have reported the extent of bacterial adhesion generally increases with increasing hydrophobicity and decreasing surface energy of abiotic surfaces for hydrophilic bacteria^26–28^. Tegoulia and Cooper^29^ utilized thiol surfaces with differing functional end-groups to study the effect of surface chemistry on *Staphylococcus aureus* adhesion and found that the bacterial adhesion was higher on the hydrophobic surfaces. However, recent studies by Pranzetti *et al*.^30^ reported, for *Marinobacter hydrocarbonoclasticus* and *Cobetia marina*, the bacterial adhesion was higher on 11-aminoundecanethiol hydrochloride surfaces, which displayed a water contact angle of 60 ± 2° (i.e., hydrophilic), than on 1-hexadecanethiol surfaces, which displayed a water contact angle of 105 ± 4° (i.e., hydrophobic).

There have been conflicting findings regarding the bacterial adhesion trends with respect to the surface chemistry due to several reasons. First, aside from the substrate hydrophobicity, substrate roughness and substrate texture, as well as material porosity^31^ and fibrousness^32^ can also alter the bacterial adhesion behavior^33–35^. To unambiguously deduce correlations between substrate chemistry and bacterial adhesion, the substrate roughness effect should be minimized. Second, experimental assays and conditions by which the bacterial adhesion have been evaluated have shown variations, such as drop-casting inoculation amplify the contribution of the gravitational and drying effects^36,37^. Third, there have been large differences in the rinsing step, which is often utilized to remove non-adherent bacteria from the substrate of interest. Owing to an introduction of the flow field during rinsing, both adherent and non-adherent bacteria experience hydrodynamic lift (i.e., proportional to the square of the velocity gradient for a spherical object on a surface) and drag force (i.e., proportional to the magnitude of the velocity)^38,39^. Depending on the interplay amidst the hydrodynamic forces and the adhesion force, the rinsing process may dislodge non-adherent bacteria only or all non-adherent and some adherent bacteria. Fourth, whether bacterial suspension is quiescent over a surface of interest or flows over it under dynamic conditions can alter the translocation of bacteria to the surface, governed by the convective-diffusion equation^40,41^. The hydrodynamic nature of bacterial suspension (i.e., static versus dynamic) becomes significant when the adhesion process is not “reaction-limited”, occurring when there is no activation barrier for adhesion (i.e., an overall attractive interaction) or when the magnitude of the repulsive activation barrier is comparable or smaller than the thermal energy, k_B_T^42,43^.

In the context of bacterial adhesion, while the majority of the existing literature have focused on the effect of surface properties on the thermodynamical/steady-state adhesion behavior, i.e., the experiments have been performed under a fixed time of bacterial exposure (sufficiently long to reach close to steady-state/equilibrium conditions)^44–48^, there is comparatively limited amount of work done about the kinetics of bacterial adhesion^49–53^. In these studies, exponential (first order) and linear (zeroth order) adhesion kinetics are the main trends observed for various permutations of surfaces and bacteria^50–53^. On the other hand, particle transport theories and studies with abiotic colloids reported the existence of power-law relationship between adhesion and time^54,55^, which has yet to be observed experimentally for bacterial systems to the best of our knowledge. In addition, the generated time-dependent adhesion data have not been sufficiently analyzed to put these into a formal reaction (“adhesion”) rate equation expressing the change in the surface concentration of bacteria in terms of reaction (“adhesion”) rate constant, reaction order, and the concentration of bacterial suspension. Such information can allow a more quantitative, direct, and reliable comparison of bacterial adhesion kinetics among researchers. This study is aimed at contributing to these aspects and bringing different perspectives to the field of microbiology. To this end, we relied on molecularly smooth substrates with precisely controlled surface chemistry and types of bacteria (i.e., *Staphylococcus aureus* and *Escherichia coli* O157:H7). Here, *S*. *aureus* and *E*. *coli* O157:H7, commonly causing human foodborne illnesses^56–58^, were selected as model bacteria in this study because they exhibit differing shapes (i.e., coccoid: round-shaped; bacilli: rod-shaped) and Gram-reactions. According to the Centers for Disease Control and Prevention (CDC) analysis, *E*. *coli* O157:H7 infections cause 73,000 illnesses, 2,200 hospitalizations, and 60 deaths each year in the United States^59^. The annual cost of illness linked to *E*. *coli* O157:H7 was estimated to 405 million dollars, including loss of productivity, medical care expenses, and mortality^60^. About 30% of the human population is colonized with *S*. *aureus*^61^. In addition, it is a leading cause of device-related infections, bacteremia and infective endocarditis, osteoarticular infections, skin and soft tissue diseases, and pleuropulmonary infections^62^. The kinetical and thermodynamical knowledge gained through this study guides the selection of surface hydrophobicity and potential combinations for reducing bacterial adhesion in applications exposed bacteria at various time intervals. In particular, this study recommends the use of surfaces and materials with high hydrophilicity and large negative zeta potential values for minimizing *S*. *aureus* and *E*. *coli* O157:H7 adhesion on them. We also note that while surfaces with large positive zeta potential values can disintegrate and rupture *E*. *coli* O157:H7, the irreversible attached bacteria reduce (consume) available surface area, making this approach unfeasible for long-term applications.

## Results and Discussion

### Characterization of substrates

**Figure 1:**
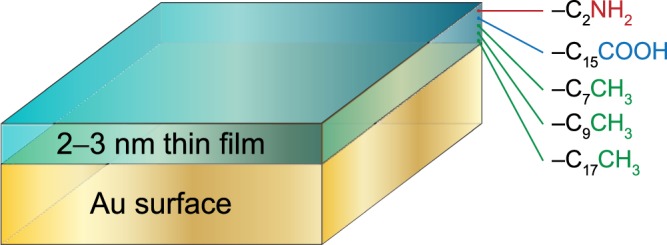
Schematic representation of substrates involving 2–3 nm thin film of varying surface chemistry used in this study. The specific ligands utilized for this purpose were 2-aminoethanethiol hydrochloride, 16-mercaptohexadecanoic acid, 1-octanethiol, 1-decanethiol, and 1-octadecanethiol (from top to bottom).

In this study, we have utilized three surfaces coated with hydrophobic long-chain hydrocarbons (1-octanethiol, 1-decanethiol, and 1-octadecanethiol), one hydrophilic surface with negative zeta potential value (16-mercaptohexadecanoic acid), and one hydrophilic surface with positive zeta potential value (2-aminoethanethiol hydrochloride) (Fig. 1). The precise characterization of the substrates is a prerequisite for deriving reliable correlations between substrate chemistry and bacterial adhesion. To this end, a variety of approaches including contact angle (goniometer) technique and streaming potential technique were used, the results of which are given in Table 1. It was found that while the surface energies of substrates with amine and carboxylic acid-terminated layers were relatively high, approximately 56 mJ/m^2^ and 53 mJ/m^2^, respectively, those of surfaces with long-chain alkyl-terminated layers ranged from 21 to 33 mJ/m^2^, decreased with increasing chain length. While the ratios of nonpolar component of surface energy to the total surface energy were 73% and 76% for hydrophilic amine-terminated (-C_2_NH_2_) and hydrophilic carboxylic acid-terminated (-C_15_COOH) surfaces, respectively, corresponding ratios were 95%, 95%, and 99% for functionalized with hydrophobic methyl-terminated surfaces, i.e., -C_7_CH_3_, -C_9_CH_3_, and -C_17_CH_3_, respectively.

**Table 1:** The list of substrates with varying types of coatings used to systematically study the influence of surface chemistry and charge on bacterial adhesion.

Surface	Contact angle of water (°)	Contact angle of DIM (°)	Nonpolar surface energy (mJ/m^2^)	Polar surface energy (mJ/m^2^)	Surface energy (mJ/m^2^)	Zeta potential (mV)	Film thickness (nm)
-C_2_NH_2_	54.5 ± 1.0	36.1 ± 0.5	41.5 ± 0.2	14.8 ± 0.5	56.3 ± 0.6	+10.3	2.4 ± 0.3
-C_15_COOH	59.3 ± 0.7	37.5 ± 0.5	40.9 ± 0.3	12.4 ± 0.3	53.3 ± 0.5	−47.6	2.9 ± 0.3
-C_7_CH_3_	92.5 ± 0.7	55.5 ± 0.6	31.2 ± 0.3	1.5 ± 0.2	32.7 ± 0.2	−22.7	2.5 ± 0.3
-C_9_CH_3_	98.6 ± 0.8	66.4 ± 0.7	24.9 ± 0.4	1.2 ± 0.1	26.1 ± 0.4	−22.9	2.9 ± 0.3
-C_17_CH_3_	108.4 ± 0.7	72.8 ± 0.6	21.4 ± 0.3	0.2 ± 0.1	21.6 ± 0.3	−14.0	3.0 ± 0.3

The zeta potential was mildly positive for amine-functionalized surfaces, and was negative with carboxylic acid- and alkyl-terminated surfaces (Table 1). While the dissociation of carboxylic acid and the protonation of amino groups can explain the charging of hydrophilic substrates, the existence of a negative zeta potential for hydrophobic surfaces is counter-intuitive as hydrophobic materials are expected to be non-dissociating and non-associating. This behavior, also observed by various other groups, was ascribed to predominately the specific organization and orientation of hydroxyl ions onto hydrophobic interfaces^63^.

To estimate the coverage of chemical groups on the functionalized surfaces accurately, secondary ion mass spectrometry (SIMS) based on coincidence counting was used^64^. These measurements indicated the surface coverage of surface functionalization was high, >92% for all chemical groups (see Supplementary Information Section 1 and Table S1). Film thickness was measured with ellipsometry and found to be in the range of 2–3 nm for all ligands.

### Characterization of bacteria

Table 2 summarizes the key structural and physicochemical properties of *S*. *aureus* and *E*. *coli* O157:H7 isolates. The round-shaped *S*. *aureus* was about three times smaller than the rod-shaped *E*. *coli* O157:H7. Static contact angle of water on both microorganisms was relatively low, indicating hydrophilic nature of their surfaces. The zeta potential was −37.1 mV and −12.7 mV for *S*. *aureus* and *E*. *coli* O157:H7, respectively. The cell wall of *S*. *aureus* involves layers of peptidoglycans that are rich in teichoic acid groups^65,66^. The measured negative value of the zeta potential for *S*. *aureus* is ascribed to the existence of anionic phosphate groups in the glycerol phosphate repeating units of teichoic acids^67^. The outer layer of *E*. *coli* O157:H7 contains mostly lipopolysaccharides^68,69^, which include phosphate groups in the inner core and polar hydroxyl groups in sugar repeating units (*N*-acetyl-D-perosamine, L-fucose, D-glucose, and *N*-acetyl-D-galactose) of the O-antigen^70,71^. Phosphate and hydroxyl groups can account for negative zeta potential of *E*. *coli* O157:H7. Observed hydrophilicity can also be attributed to the charged groups and polar groups.

**Table 2:** The key structural and interfacial characteristics of *S*. *aureus* and *E*. *coli* O157:H7 used in this study.

Bacteria	*S*. *aureus*	*E*. *coli* O157:H7
Type	Gram-positive	Gram-negative
Dimensions (µm)	*D*: 0.72 ± 0.04 (round-shaped)	*W*: 0.98 ± 0.15; *L*: 2.34 ± 0.19 (rod-shaped)
Contact angle of water (°)	33.2 ± 1.8	30.7 ± 2.8
Contact angle of DIM (°)	40.1 ± 2.2	42.0 ± 1.0
Surface energy (mJ/m^2^)	67.9 ± 1.6	67.1 ± 1.2
Zeta potential (mV)	−37.1 ± 0.6	−12.7 ± 0.5
Diffusion coefficient (m^2^/s)	3.0 × 10^−13^	1.8 × 10^−13^

### Bacterial adhesion on hydrophobic substrates

**Figure 2:**
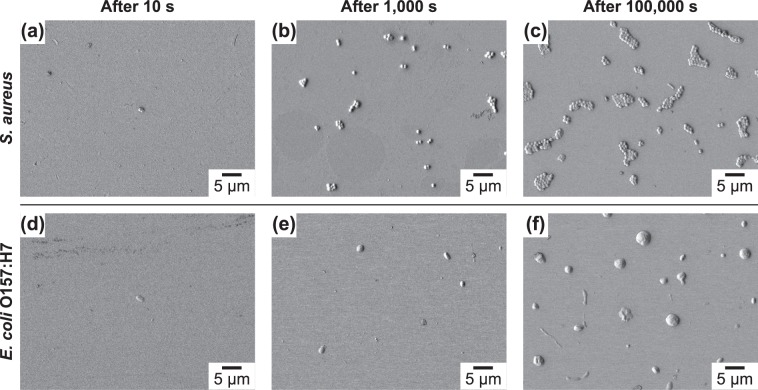
SEM micrographs showing (**a**–**c**) *S*. *aureus* and (**d**–**f**) *E*. *coli* O157:H7 adhesion on hydrophobic substrates (i.e., 1-decanethiol) with a negative zeta potential as a function of time (10 s, 1,000 s, and 100,000 s).

Scanning electron microscope (SEM) micrographs of hydrophobic substrates upon inoculation with and adhesion of *S*. *aureus* and *E*. *coli* O157:H7 are shown in Fig. 2. Based on these data, several observations can be made: First, as expected, the number of adherent bacteria increases with increasing inoculation/contact time for both Gram-positive and Gram-negative bacterial microbes. Second, there was no indication of bacterial multilayer formation during the initial stages of adhesion. Third, from a physicochemical perspective, the distribution of the bacteria on the substrate was not uniform as evidenced by the existence of bacterial clusters (microcolonies) rather than evenly spaced individual bacteria for both bacterial microbes. Fourth, while microcolonies involved well-defined bacterial divisions for *S*. *aureus*, the bacillus (rod-shaped) morphology and bacterial boundaries within the clusters were not detectable for *E*. *coli* O157:H7. Clusters with non-discrete divisions, which appear to be a dried viscous fluid, can be interpreted as bacteria covered with the extracellular polymeric substances (EPS) secreted by *E*. *coli* O157:H7 on hydrophobic surfaces.

### Bacterial adhesion on hydrophilic substrates

**Figure 3:**
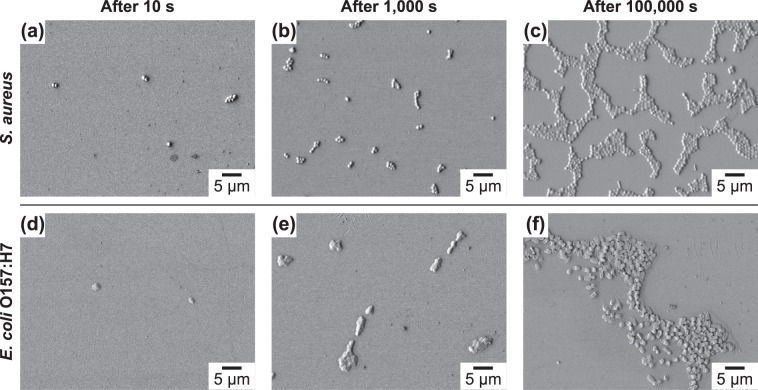
SEM micrographs showing (**a**–**c**) *S*. *aureus* and (**d**–**f**) *E*. *coli* O157:H7 adhesion on hydrophilic surfaces with a positive zeta potential (i.e., modified with 2-aminoethanethiol hydrochloride) as a function of time (10 s, 1,000 s, and 100,000 s).

Figure 3 demonstrates the adhesion behavior of *S*. *aureus* and *E*. *coli* O157:H7 on a hydrophilic substrate with a positive surface potential. The extent of adhesion on these substrates was greater than on hydrophobic substrates for both Gram-positive and Gram-negative microorganisms. Similar to observations from hydrophobic surfaces analyses, the tendency to form microcolonies and bacterial monolayers rather than multilayers were also observed in the initial stages of bacterial adhesion for both microorganisms. However, as opposed to on hydrophobic surfaces, bacillus (rod-shaped) morphology and bacterial boundaries within the clusters were detectable for *E*. *coli* O157:H7 on hydrophilic surfaces. This change may be ascribed to the better spreading of EPS on hydrophilic surfaces due to the polar nature of EPS components.

Intriguingly, the lysis of *E*. *coli* O157:H7 cell wall was observed on the substrates with a positive zeta potential (Fig. 3f) (see Supplementary Information Fig. S1 for high magnification images). It is important to note that all SEM micrographs were captured using the same method and analysis conditions, supporting the substrate specific lysis effects.

**Figure 4:**
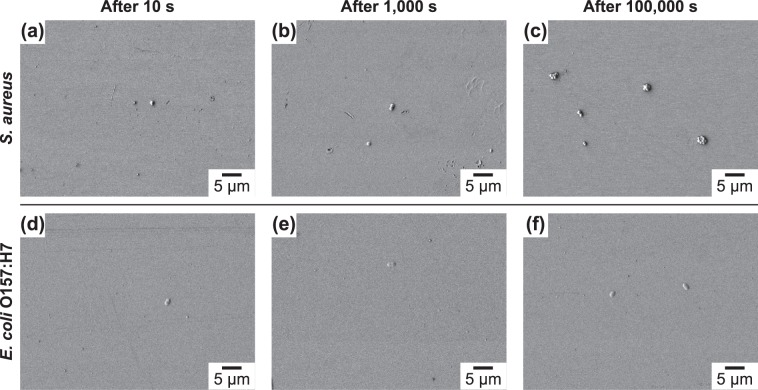
SEM micrographs showing (**a**–**c**) *S. aureus* and (**d**–**f**) *E*. *coli* O157:H7 adhesion on hydrophilic substrates (i.e., 16-mercaptohexadecanoic acid) with a negative zeta potential as a function of time (10 s, 1,000 s, and 100,000 s).

In comparison to the hydrophilic substrates with a positive surface potential, the hydrophilic substrates with a negative zeta potential displayed significant reductions in the extent of bacterial adhesion for both *S*. *aureus* and *E*. *coli* O157:H7 (Fig. 4). This finding points out the importance of electrostatic interactions in the context of bacterial adhesion. The distribution of microcolonies on substrates with positively charged and negatively charged functional groups were similar. Also, no multilayer formation was observed.

### Discussion and modeling of bacterial adhesion trends

**Figure 5:**
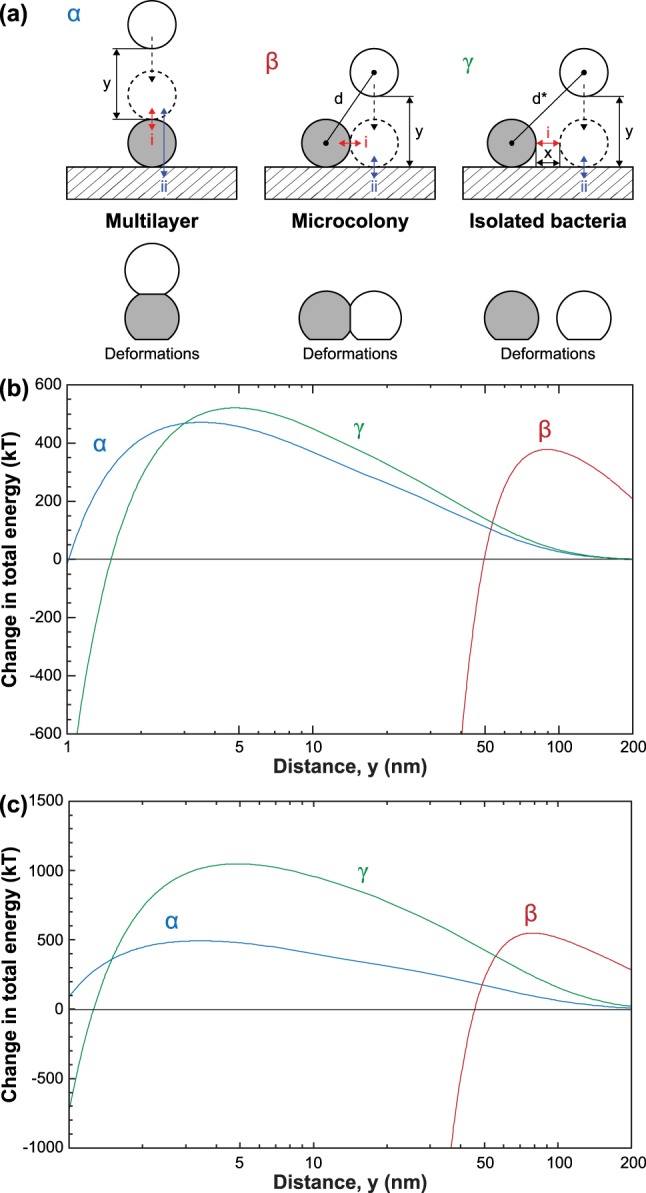
The comparison of the change in the interaction potential for the system of two coccoid (round-shaped) bacteria and a substrate under three different configurations. (**a**) In configuration α, the other bacterium approaches to the adherent bacteria from the top; in configuration β, the other bacterium approaches to next to the adherent bacteria in a vertical fashion; and in configuration γ, the other bacterium approaches to the adherent bacteria from far away distance, in a vertical fashion. The corresponding changes in the total energy as a function of distance (separation) for (**b**) a hydrophobic (-C_9_CH_3_) and (**c**) a hydrophilic substrate (-C_15_COOH).

To explain the physicochemical driving forces behind the formation of bacterial microcolonies and monolayers rather than multilayers, we calculated and compared the change in the interaction potential for the system of two round-shaped *S*. *aureus* cells and a substrate (hydrophobic: -C_9_CH_3_ and hydrophilic: -C_15_COOH) in three different configurations, after a bacterial cell pre-adheres onto the substrate: (α) the other cell approaches to the adherent cell from the top in an aligned fashion; (β) the other cell approaches vertically from off-center; and (γ) the other cell approaches to the adherent bacteria from a long distance away, in a vertical fashion (Fig. 5a). The interaction potential was calculated by considering van der Waals interactions of deforming bacterium, electrostatic double-layer interactions, and elastic energy owing to the deformation of bacterium (see Supplementary Information Section 2). In this analysis, we used the Lifshitz theory to estimate the Hamaker constant via the dielectric and refractive index values (see Supplementary Information Table S2). For any system, thermodynamics dictates that a lower energy corresponds to a more favorable configuration. For the hydrophobic substrate, as can be seen from Fig. 5b, configurations β and γ are energetically more favorable than configuration α for separations below ~53 nm and ~4 nm, respectively. At the molecular level, bacteria are in contact with the substrate when the distance between them is comparable with the van der Waals radii of carbon, oxygen, and hydrogen, i.e., building blocks of bacteria wall and coating on the substrate. At the theoretical molecular contact (i.e., cutoff distance: 0.165 nm)^72^, the change in the energy of the system, ΔE, for configuration α, ΔE_α_ is about −8.5 × 10^3^ kT as opposed to being ΔE_β_ = −42.4 × 10^3^ kT for configuration β and ΔE_γ_ = −34.0 × 10^3^ kT for configuration γ. In reality, however, these differences are expected to be much smaller because the Born repulsion becomes significant at ultrasmall separations when the electron clouds of two surfaces start to overlap. These findings can further be explained as follows: as van der Waals interactions are strongly dependent on distance and the distance between the incoming bacterial cell and substrate is greater than bacterial size (i.e., ~700 nm) for configuration α, the favorable interactions are primarily due to the van der Waals attractions between two bacteria. On the other hand, in configuration β, both bacterium-bacterium and bacterium-substrate interactions contributes to the minimization of free energy of the system while van der Waals interactions between substrate and bacterium are the main favorable energy term in configuration γ. Given that the van der Waals interactions are body forces and the volume of the substrate is much larger than bacteria, van der Waals interactions between two bacteria is smaller than that between a bacterium and a substrate, i.e., making configuration γ more favorable than configuration α. Clearly, the additional attractive interactions arising when two bacteria near each other makes configuration β the most favorable one.

It is also important to note that the energy diagram is derived for the interaction between a sphere and a flat wall, which is valid for *S*. *aureus*. The energy calculation for *E*. *coli* O157:H7 can be done by introducing the Derjaguin correction factor^73^ and by considering the Boltzmann probability factor and obtaining a weighted average across all orientations^74^. However, since the aspect ratio is relatively small (i.e., ~2) for *E*. *coli* O157:H7, large deviations in interaction potential between spherical and rod-shaped bacteria are not expected. However, since the zeta potential of *E*. *coli* O157:H7 surfaces are three times smaller, the electrostatic effects will be less significant for the case of *E*. *coli* O157:H7 compared to *S*. *aureus*.

Similar trends were also observed for the hydrophilic substrate with the negative zeta potential (Fig. 5c): the minimization of free energy favors configuration β the most, followed by configuration γ, and configuration α the least at the molecular contact (adhesive contact). However, for the hydrophilic case, a drastic increase in the energy barrier for adhesion is observed in configuration β, which is consistent with the experimental observations of very small number of bacteria on this substrate (Fig. 4). Slight differences in the magnitude of the energy change in each configuration is primarily due to the slight increase in the Hamaker constant arising from the increased polarization of the substrate and increase in the magnitude of the surface potential of the substrate. For the positively charged substrate (not shown), the existence of attractive double-layer interactions translates into the absence of energy barrier. However, given that the van der Waals interactions dominates over electrostatics at the molecular contact, no change in the preferential configuration will be observed.

Aside from intermolecular interactions, a combination of factors may contribute to the formation dynamics of microcolonies: the secretion of EPS, such as polysaccharides, proteins, lipids, and humic substances may modify the surface chemistry of abiotic surfaces in the proximity of adherent bacteria, enhancing the cluster formation^75^. Regarding the chemical communications, quorum sensing is a mechanism of bacterial gene regulation that is based on the synthesis and release of autoinducers, which are diffusible chemical signals. At relatively high bacterial population densities, these autoinducers can locally accumulate and trigger population-wide changes in gene expression, which can modulate biofilm formation, genetic competence, symbiosis, motility, and the production of virulence factors^76^. However, this effect is not expected to be play any role considering the timescale of our studies, i.e., much shorter than time-scales needed for genetic modifications. As a second means of chemical communications, chemotaxis should be considered: chemotactic bacteria can sense and respond to chemical gradients through receptor molecules embedded in the cell membrane^77^. *E*. *coli* was reported to respond to spatiotemporal chemical gradients by actively swimming through them^78,79^. Bales *et al*.^80^ carried out glycosyl composition analysis of exopolysaccharides from *E*. *coli* O157:H7 and found that it contains glucose, n-acetyl-galactosamine, fucose, and mannose at high concentrations, all of which are known chemoattractants for *E*. *coli*^81^. Hence, for *E*. *coli*, the excretion and presence of exopolysaccharides can partly explain the chemotaxis-assisted formation of bacterial clusters. On the other hand, due to the absence of flagella, *S*. *aureus* is considered as a non-motile microorganism^82,83^. Therefore, the chemotactic effects are expected to be minimal for *S*. *aureus*. It is also important to note that thiol molecules are strongly affixed to the substrate and not solubilized and distributed in the solution, which is likely to prevent bacterial biosensing via receptor molecules unless bacteria come into contact with the substrates.

Regarding the formation of microcolonies without any visible cellular boundary on hydrophobic substrates but not on hydrophilic ones for *E*. *coli* O157:H7 (cf. Figs 2f, 3f and 4f), we consider the components of EPS and its wetting characteristics. Glycosyl composition analysis by Bales *et al*.^80^ reported EPS of *E*. *coli* O157:H7 mainly contains glucose (36.8%), *N*-acetyl-galactosamine (26.8%), fucose (22.6%), and mannose (9.8%). The solubility of mannose, glucose, and fucose is 745 mg/mL, 909 mg/mL, and 985 mg/mL, respectively^84–86^ indicating a polar/hydrophilic nature of EPS. The interfacial energy mismatch between hydrophilic EPS and hydrophobic substrates can account for the disappearance of cellular boundaries for *E*. *coli* O157:H7 microcolonies. On the other hand, cellular boundaries of microcolonies were distinct on all substrates for *S*. *aureus* microcolonies (cf. Figs 2c, 3c and 4c). The spreading/wetting of staphylococcal EPS on both hydrophobic and hydrophilic substrates is indicative of its amphiphilic nature, which can be imparted in EPS via amphiphilic peptides such as phenol-soluble modulins produced by *S*. *aureus*^87^.

Regarding the lysis (rupture) of cellular membrane of *E*. *coli* O157:H7 on hydrophilic substrates functionalized with positively charged amines (Figs 4f and S1), the membrane instabilities induced by electrostatic interactions and interactions can be considered. Quaternary amines have been known to disrupt bacterial cellular membrane through electrostatic interactions where the positively charged amine head group attaches to the negatively charged bacterial membrane, proceeding with the permeation of side chains into the intramembrane region and leakage of cytoplasmic material^88^. In our system, the amine groups were chemically bound to the substrate and pointing outward. Hence, while it is not possible for these molecules to be freely diffused and absorbed into/by the bacterial cells, attractive electrostatic interactions can still pull the cell wall and form a dipole at the substrate/bacterium interfaces. The existence of such dipoles is shown to be enough to destabilize and lyse liposomes/lipid vesicles on oppositely charged surfaces^89^, which can also be the reason for the observed bacterial lysis in this work. The lack of apparent lysis in Gram-positive bacteria (i.e., *S*. *aureus*) can be explain by the existence of a thicker cell wall and lack of an external surface membrane.

### Detailed analysis of adsorption kinetics of bacteria and deduction of adhesion rate parameters

**Figure 6:**
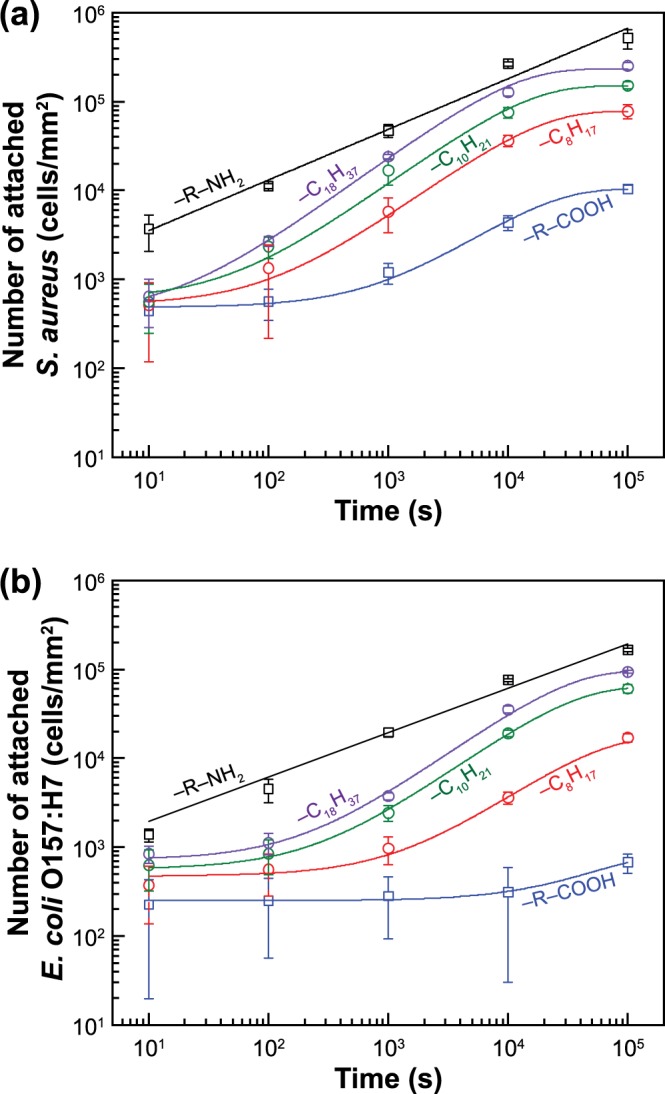
Numbers of adherent bacterial cells on substrates with systematically varying surface chemistry as a function of time for (**a**) *S*. *aureus* and (**b**) *E*. *coli* O157:H7. The error bars represent standard deviation from the mean.

The enumeration of adherent bacteria using a large number (n > 10) of SEM micrographs allowed us to precisely quantify the bacterial adhesion in a time-resolved fashion (Fig. 6). Overall, the extent of bacterial adhesion was the largest on surfaces with amine-functionalization, followed by hydrophobic substrates with a decreasing hydrophobicity, and carboxylic acid functionalized substrates. For a given surface and exposure time, count of adhering *S*. *aureus* was greater than that for *E*. *coli* O157:H7 (*p* < 0.05). A further analysis of micrographs revealed, for both *S*. *aureus* and *E*. *coli* O157:H7, the initial stage of bacterial adhesion process followed a power-law behavior in the case of the hydrophilic substrates with a positive zeta potential. On the other hand, the adhesion dynamics followed an exponential behavior for the hydrophilic and hydrophobic substrates with a negative zeta potential. Namely, there was a transition from a power-law to an exponential behavior as the intermolecular interactions between substrate and bacterium changed from attractive to repulsive.

The exponent of the power law for amine-terminated surfaces was 0.57 ± 0.06 (r^2^ = 0.959) and 0.49 ± 0.04 (r^2^ = 0.884) for *S*. *aureus* and *E*. *coli* O157:H7, respectively. An adhesion kinetics with a square root dependence on time, i.e., ∝*t*^1/2^, is indicative of a diffusion-controlled process, which occurs when there is no energy barrier for adhesion. Alternatively, the activation energy of adhesion is comparable with the thermal energy, k_B_T where k_B_ is the Boltzmann constant and T is the absolute temperature^90–92^. Considering the zeta potential of bacteria (−37.1 mV for *S*. *aureus* and −12.7 mV for *E*. *coli* O157:H7) and amine-terminated thiol surfaces (+10.3 mV), the double-layer interactions are attractive for both cases, which can elucidate the observed adhesion kinetics trends.

For the hydrophilic substrate with a negative zeta potential (i.e., carboxylic acid-terminated thiol surfaces) and hydrophobic substrates (i.e., methyl-terminated thiol surfaces) with long-chain hydrocarbons having an “apparent” negative zeta potential, the adhesion kinetics followed an exponential behavior, i.e., ∝*e*^*−t*/*τ*^, indicating the bacterial adhesion was a first-order process. The time constant (*τ*) increased with decreasing substrate hydrophobicity and was larger for hydrophilic substrate compared to hydrophobic substrates (Table 3) (p < 0.05). For a given substrate, the time constant of *E*. *coli* O157:H7 adhesion was two to four times greater than that of *S*. *aureus* adhesion, meaning that *S*. *aureus* attaches on substrates at a faster rate (number wise). For substrates with a positive zeta potential, since the adsorption process is diffusion-limited, rather than the reaction time constant, it is appropriate to calculate the diffusion time constant:

1 $${\tau }_{d}\approx \frac{{L}_{c}^{2}}{4D}$$

here, *L*_*c*_ is the average distance between bacteria, which is governed by the concentration of bacterial suspension and equal to 1.2 × 10^−5^ m in our experiments while *D* is the diffusion coefficient of bacteria, which can be estimated from the Stokes-Einstein equation based on the bacterial size (Table 2). For both *S*. *aureus* and *E*. *coli* O157:H7, the diffusion time constant was about two-orders of magnitude smaller than the “reaction” time constant, indicating that the adhesion on hydrophilic substrate with a positive zeta potential is much faster compared to hydrophilic and hydrophobic substrates with a negative zeta potential.

**Table 3:** The calculated time constant by analyzing Fig. 6.

Substrate	Time constant (s) for *S*. *aureus*	Time constant (s) for *E*. *coli* O157:H7
-C_2_NH_2_	112.4 ± 8.8	187.4 ± 33.9
-C_15_COOH	18610 ± 3093	74415 ± 90733
-C_7_CH_3_	15680 ± 1395	47210 ± 9614
-C_9_CH_3_	12590 ± 2982	29270 ± 2328
-C_17_CH_3_	9757 ± 1777	26890 ± 4387

The parameters were obtained based on the exponential-decay characterizing a first-order adhesion process all substrates except amino-terminated one, where the diffusion time constant was calculated from Equation 1.

### Implications of research findings

While we establish an adhesion preference trend for aqueous *S*. *aureus* and *E*. *coli* O157:H7 suspensions, it is important to underline that experimental surfaces were molecularly smooth with a root-mean-square roughness less than 2 nm. The substrate texture can also alter the adhesion characteristics as it can provide crevices, dips, and valleys for bacteria to interact not only vertically but also horizontally with the substrates^93^. Engineered and naturally occurring surfaces often involve multiple roughness length scales, further complicating the effect of the bacterial adhesion process. With this study, we aim to de-convolute chemistry and roughness factors to gain deeper insights into the influence of surface chemistry on the adhesion of experimental bacterial organisms. Furthermore, the use of static rinsing step three times after the bacterial inoculation step is extremely crucial in distinguishing the drying effects (i.e., the attachment of bacterial to surfaces induced by evaporation of water) from the surface chemistry effects governed by intermolecular forces. In fact, hydrophilic surfaces with a negative zeta potentials can exhibit significant bacterial adhesion when the rinsing step is not used^94^. This is mostly because bacteria suspended in water droplet on a surface, although they may not adhere onto the surface, are hydrodynamically restricted by the droplet. With gradual evaporation of water on the surface, suspended bacteria are destined to be localized on the surfaces. For cases of an external force field such as gravitational forces and pressure difference resulting in a flow field with a convective-diffusion bacterial dynamics, the interplay among the rate of deposition, rate of evaporation, and rate of flow of bacterial suspension can lead to further complications in the adhesion trends with respect to surface chemistry^17^.

In essence, herein we gained further insights into how the surface chemistry influences the dynamics of bacterial adhesion using *S*. *aureus* and *E*. *coli* O157:H7 and model surfaces with precisely controlled surface chemistry and thickness. Bacterial adhesion was greatest on hydrophilic substrates with positive surface charge characteristics, followed by hydrophobic substrates with negative surface charge characteristics while increasing hydrophobicity, and smallest on hydrophilic substrates with negative surface charge characteristics. The time constant of adhesion was about two to four times greater for *E*. *coli* O157:H7 compared to *S*. *aureus*, indicating a slower number attached per unit time for *E*. *coli* O157:H7 but a comparable mass attached per unit time. A transition from a power-law to an exponential dependence on time was observed upon changing from hydrophilic substrates with a positive zeta potential to hydrophilic and hydrophobic substrates with a negative zeta potential. In addition, a model relying on intermolecular forces was used to explain the formation of microcolonies rather than isolated and evenly spaced bacteria on surfaces at the initial stages of bacterial adhesion. It was found that aside from the standard DLVO interactions, the deformation energy must be considered to properly explain the bacterial adhesion trends and the formation of microcolonies. Only surfaces with positively charged groups led to the lysis of *E*. *coli* O157:H7 but not the lysis of *S*. *aureus* upon adhesion, which are attributed to the electrostatic disruption of thinner cell membrane of *E*. *coli* O157:H7.

## Methods

### Surface preparation methods

Gold (Au) coated plates with thickness of 0.5 mm cut into 10 mm × 10 mm were first rinsed with acetone (Avantor Performance Materials, Inc., Center Valley, PA, USA). Subsequently, surfaces were immediately rinsed with absolute 200 proof ethanol (Koptec, King of Prussia, PA, USA) and dried under a stream of nitrogen gas (N_2_; Brazos Valley Welding Supply, Inc., Bryan, TX, USA) before use. Linear-chain thiols were used to prepare hydrophilic and hydrophobic substrates with systematically varying wetting characteristic. 2-aminoethanethiol hydrochloride (-C_2_NH_2_), 16-mercaptohexadecanoic acid (-C_15_COOH), 1-octanethiol (-C_7_CH_3_), 1-decanethiol (-C_9_CH_3_), and 1-octadecanethiol (-C_17_CH_3_) were purchased from Sigma-Aldrich Co. (St. Louis, MO, USA) and used without purification. Thiol solution was prepared by dissolving thiol of interest in ethanol at 5 mM concentration via 10 min of sonication at room temperature (23 °C). Afterwards, the gold plates were submerged in thiol solution of interest at room temperature to form a stable coating on the surface with a similar thickness. The immersion time was varied to make sure that the resultant coating thickness is the same for all ligands, in the range of 18 to 24 h.

### Contact angle measurements

To gain insights into the interfacial characteristics of various thin films deposited on gold surfaces, the static water and diiodomethane (DIM) contact angles were monitored on these surfaces using the sessile drop technique^95^. In these experiments, as water source, Milli-Q water with a resistivity of 18.2 MΩ/cm (at 25 °C) was utilized. Diiodomethane was purchased from Sigma-Aldrich Co. (St. Louis, MO, USA) and used as-received. From the contact angle data, surface energy of substrates with varying chemical functionalization was calculated. Contact angle measurements were also conducted on bacteria layers. We have followed the prior studies describing the measurement of water contact angle on alive *S*. *aureus* and *E*. *coli* O157:H7 layer collected on a microfilter^96,97^. Cells were collected on nitrocellulose filters (Millipore, 0.8 µm pore size; EMD Millipore Corp., Billerica, MA, USA), and 100 µL of phosphate buffer was dropped on the filter. The water droplet was slowly moved towards the bacterial layer residing on the filter, and upon contact, the image was captured using a high-resolution camera. For both cases, reported contact angles were obtained by averaging four independent measurements at room temperature. The analysis of contact angles was carried out via ImageJ software (National Institutes of Health, Bethesda, MD, USA) with the aid of LBADSA plug-in^98^.

### Secondary ion mass spectrometry (SIMS) measurements

SIMS measurements were performed to determine the coverage of chemical functionalization on the substrates. The C_60_ SIMS measurements were carried out with a custom-built SIMS instrument coupled to a time-of-flight mass analyzer^99^. The instrument used in these studies is equipped with a C_60_ effusion source capable of producing C_60_^2+^ projectiles with total impact energy of 50 keV. The SIMS analysis of the samples was conducted in the super-static regime (<0.1% of the analyzed surface is impacted) in the event-by-event bombardment-detection mode, where a single primary projectile (C_60_^2+^) impacted on the surface, and the secondary ions were collected and analyzed before subsequent primary projectiles impacting the surface^100^.

### Streaming potential measurements

In the streaming potential experiments, miniature streaming potential apparatus was used as described elsewhere^101^. The zeta potentials were calculated from measured streaming potential values via the Smoluchowski equation^102^. Ionic strength effects were examined with 1, 10, and 100 mM KCl solutions. The salts used in the experiments were of analytical grade (Merck KGaA, Darmstadt, Germany). All salt solutions were prepared using ultrapurified water (Milli-Q Advantage A10; EMD Millipore Corp., Billerica, MA, USA). Two silver/silver chloride (Ag/AgCl) electrodes were utilized to measure the streaming potential through an electrolyte solution flowing through the apparatus under constant hydrostatic pressure controlled by a programmable syringe pump (Fusion 200, Chemyx Inc., Stafford, TX, USA).

### Bacterial cultures and preparation for surface adhesion experiments

*Staphylococcus aureus* (ATCC 13368) and *Escherichia coli* O157:H7 (ATCC 700728) were revived from −80 °C storage in the Department of Animal Science Food Microbiology Laboratory (Texas A&M University, College Station, TX, USA) by duplicate identical passages in tryptic soy broth (TSB; Becton, Dickinson and Co., Sparks, MD, USA) followed by incubation (18 h at 37 °C). The final populations of *S*. *aureus* and *E*. *coli* O157:H7 in the growth medium following incubation ranged from 8.6 to 9.0 log_10_ CFU/mL. For bacterial pathogen inoculation onto substrates, thiol-coated gold surfaces were submerged in 9.0 mL of a bacterial suspension at room temperature for 10, 100, 1,000, 10,000, and 100,000 s. Samples coated with various functional groups were then lifted gently from bacterial medium in a smooth vertical motion. Afterwards, samples were rinsed with sterile Milli-Q water three times to dislodge weekly bound cells, and then moved to sterile Petri dishes in order to assay bacterial adhesion on thiol surfaces. A special attention was paid to ensure that the rinsing did not introduce any significant flow field or shear stress around the sample by immersing and removing the samples from Milli-Q water very slowly (i.e., at a velocity of 1 cm/min). All experiments were carried out in the Class II, Type A biological safety cabinet under biosafety level-2 containment conditions. Inoculation experiments were replicated four times.

The direct enumeration of attached bacteria on thiol surfaces that were dipped in the inoculum for 10, 100, 1,000, 10,000, and 100,000 s was conducted using scanning electron microscope (SEM, JSM-7500F; JEOL, Tokyo, Japan). Prior to SEM imaging, bacteria were inactivated by acrolein (Sigma-Aldrich Co., St. Louis, MO, USA) treatment and a thin layer (15 nm) of platinum-palladium (Pt-Pd) alloy film was deposited on the sample surfaces to prevent charging of the specimen. Micrographs obtained via SEM were examined using ImageJ software to quantify the adhesion of *S*. *aureus* and *E*. *coli* O157:H7 to substrates covered with varying functional groups. For statistical reliability, at least ten different areas of 100 mm × 100 mm (i.e., scan area larger than 100,000 mm^2^) from the three different samples of the same type of thiol surface were observed^103^.

### Statistical analysis

As a first step, microbiological data were log_10_-transformed. Then, one-way and two-way analysis of variance (ANOVA) with Tukey’s post-hoc means separation test was performed to identify statistically significant differences in the bacterial adhesion density and rates between substrate types for the two experimental bacterial microbes with *p* = 0.05. All statistical analyses were carried out using Origin 8 software (OriginLab Co., Northampton, MA, USA).

## Electronic supplementary material

Supplementary Information
