# Supplementary material for: The influence of surface chemistry on the kinetics and thermodynamics of bacterial adhesion
Source: Sci Rep. 2018 Nov 22;8:17247. doi: 10.1038/s41598-018-35343-1 (PMC6250697; doi:10.1038/s41598-018-35343-1)
Supplement: Supplementary file 1 — Supplementary Information [file 41598_2018_35343_MOESM1_ESM.pdf]

Supplementary Information

The influence of surface chemistry on the kinetics and thermodynamics of bacterial adhesion

Jun Kyun Oh[1], Yagmur Yegin[2], Fan Yang[3], Ming Zhang[4], Jingyu Li [4], Shifeng Huang[4], Stanislav V. Verkhoturov[3], Emile A. Schweikert[3], Keila Perez-Lewis[5], Ethan A. Scholar[1], T. Matthew Taylor[5], Alejandro Castillo[5], Luis Cisneros-Zevallos[6], Younjin Min[4,*] & Mustafa Akbulut[1,7,*]

[1]Artie McFerrin Department of Chemical Engineering, Texas A&M University, College Station, Texas, 77843, USA
[2]Department of Nutrition and Food Science, Texas A&M University, College Station, Texas, 77843, USA
[3]Department of Chemistry, Texas A&M University, College Station, Texas 77843, USA
[4]Department of Polymer Engineering, University of Akron, Akron, Ohio, 44325, USA
[5]Department of Animal Science, Texas A&M University, College Station, Texas, 77843, USA
[6]Department of Horticultural Sciences, Texas A&M University, College Station, Texas, 77843, USA
[7]Department of Materials Science and Engineering, Texas A&M University, College Station, Texas, 77843, USA

[*]Correspondence and requests for materials should be addressed to Y.M. (email: ymin@uakron.edu) or to M.A. (email: makbulut@tamu.edu).

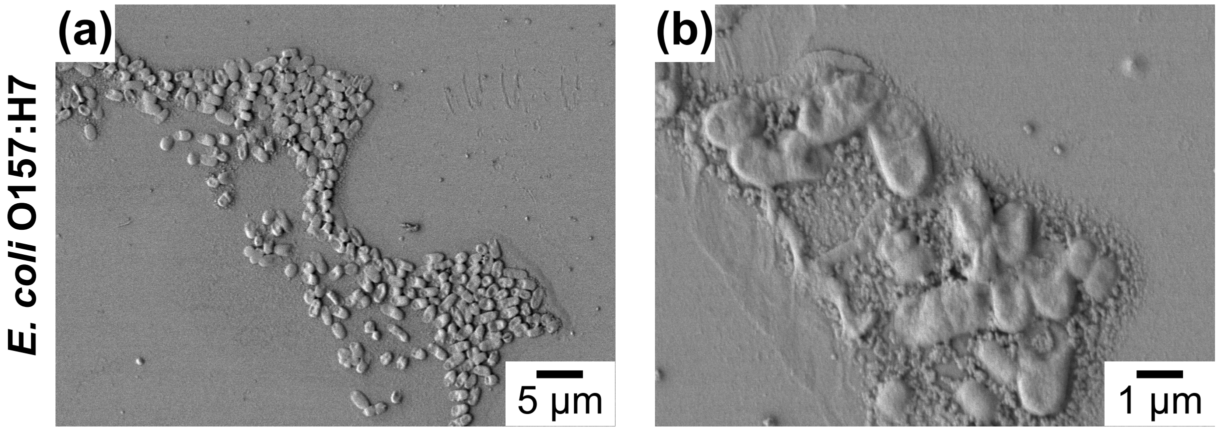

Figure S1. (a) SEM micrograph showing the lysis of *E. coli* O157:H7 cell wall. (b) High magnification SEM micrograph.

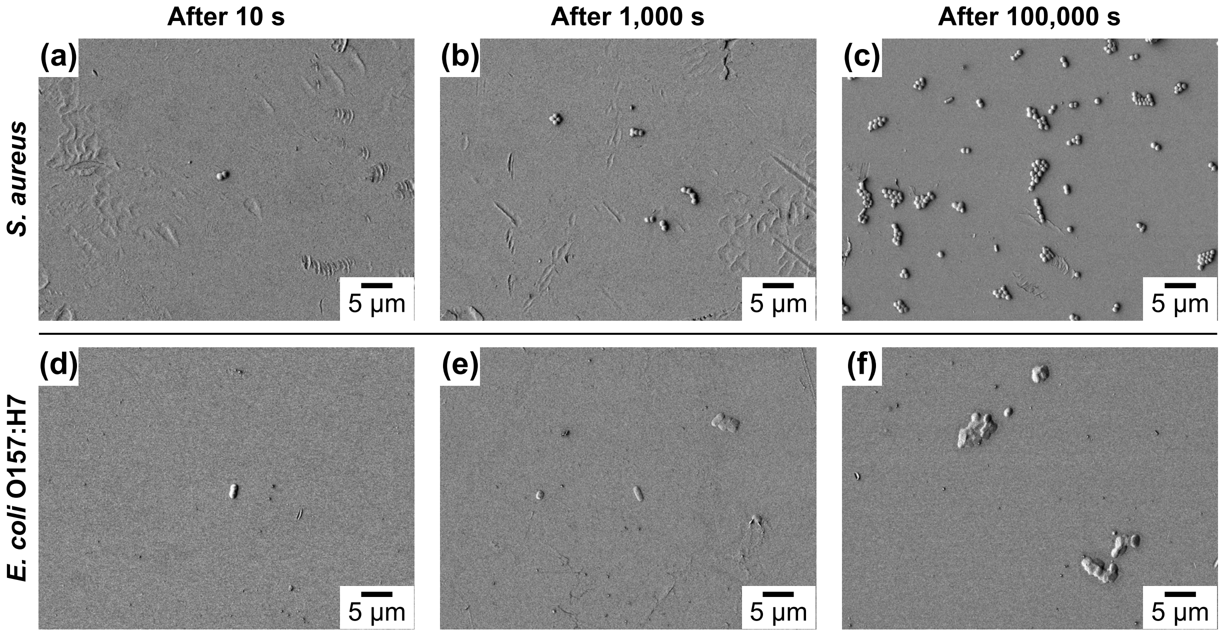

Figure S2. SEM micrographs showing (a)–(c) *S. aureus* and (d)–(f) *E. coli* O157:H7 adhesion on hydrophobic substrates (i.e., 1-octanethiol) with a negative zeta potential as a function of time (10 s, 1,000 s, and 100,000 s).

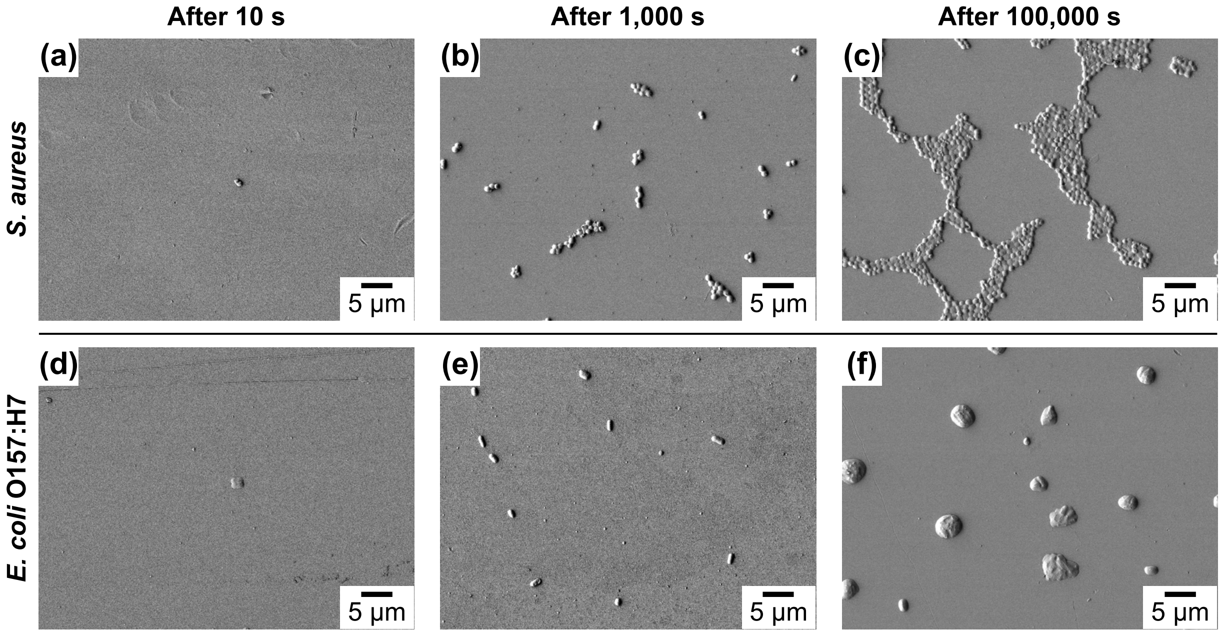

Figure S3. SEM micrographs showing (a)–(c) *S. aureus* and (d)–(f) *E. coli* O157:H7 adhesion on hydrophobic substrates (i.e., 1-octadecanethiol) with a negative zeta potential as a function of time (10 s, 1,000 s, and 100,000 s).

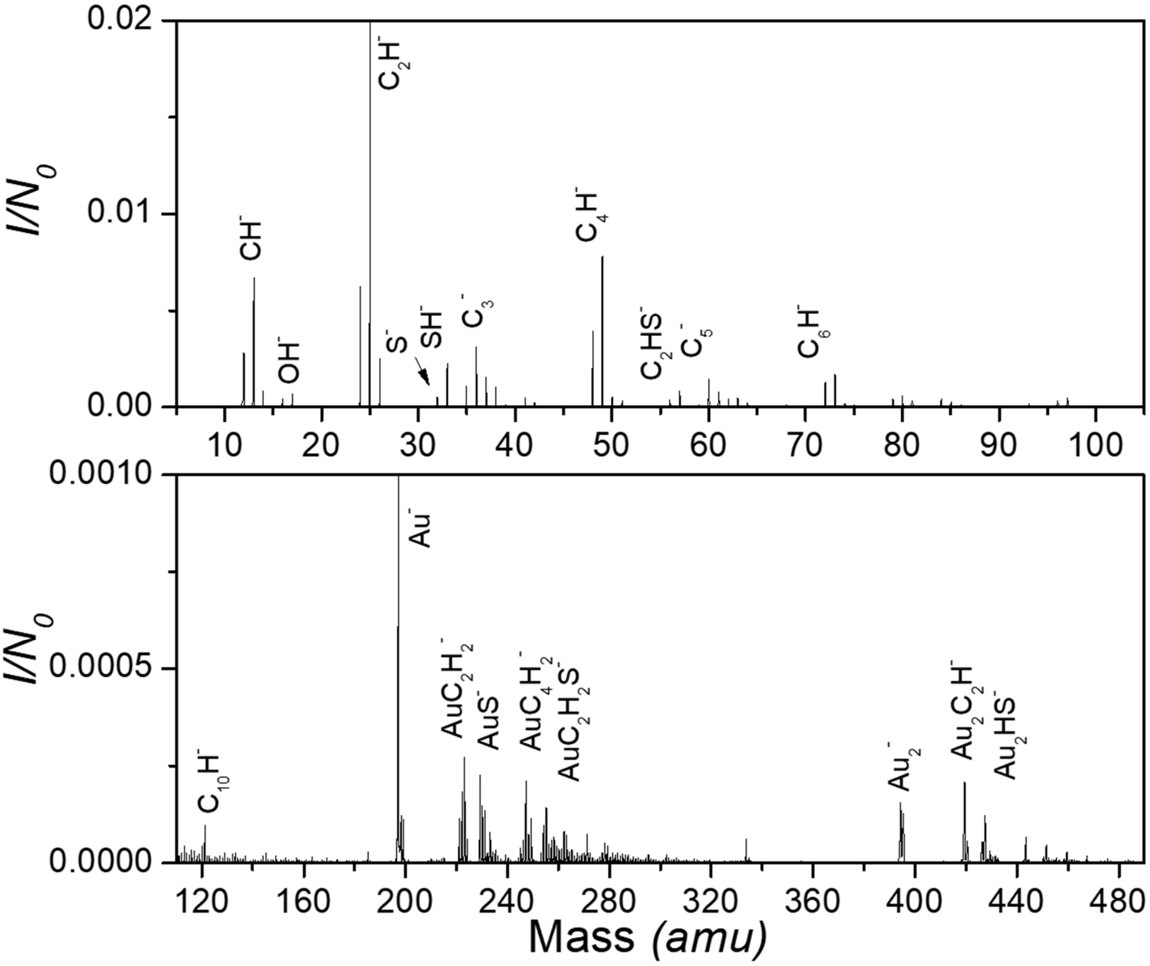

Figure S4. SIMS mass spectra of 1-octadecanethiol surfaces.

1. Secondary ion mass spectrometry (SIMS) analysis of coating layer. Fig. S4 shows the negative secondary ion mass spectra of 1-octadecanethiol adsorbed on gold (Au)-coated glass slides. The ion peaks at m/z 33 (SH^-), 57 (C_2HS^-), and 229 (AuS^-) originated from the 1-octadecanethiol. Carbon-based cluster ions, C_n^- and C_nH^-, ($1 \leq n \leq 10$) are fragmentations from both 1-octadecanethiol and C_{60} projectiles. All other SIMS spectra of linear-chain thiols are similar to those of 1-octadecanethiol adsorbed on gold surfaces.

To estimate the surface coverage of 1-octadecanethiol, a statistical method based on coincidence counting was used[1]. The relevant co-emitted secondary ions (SIs) are quantified with a correlation coefficient, Q, which is defined by:

$$Q_{A,B} = \sum_{x_A} \sum_{x_B} x_A x_B P_{(x_A x_B)} / \sum_{x_A} x_A P_{(x_A)} \sum_{x_B} x_B P_{(x_B)} \qquad \text{(S1)}$$

where x_A and x_B are the numbers of SIs simultaneously detected for species A and B, respectively; $P_{(xAxB)}$ is the probability distribution of the number of ions A and B that were detected simultaneously, and $P_{(xA)}$ and $P_{(xB)}$ are the probability distributions of separately detected ions A and B, respectively. The two types of co-emitted ions, A and B, originating from the same compound (in the present case, both emitted from fluorine segments) are assumed to have a correlation coefficient ($Q_{A,B}$) of the unity. $Q_{A,B}$ is expressed as follows:

$$Q_{A,B} = \frac{Y_{A,B}}{Y_A \times Y_B} = 1 \tag{S2}$$

where $Y_{A,B}$ is the coincidental yield of simultaneously detected ions A and B; and Y_A and Y_B are the SI yields of detected ions A and B, respectively. The $Q_{A,B}$ of the ions is equal to 1 when they originate from the same compound. Moreover, the coincidental yield $Y_{A,B}$ is defined as follows:

$$Y_{A,B} = \frac{N_{A,B}}{N_e} \tag{S3}$$

where N_e is the effective number of impacts on a specific specimen and $N_{A,B}$ is the number of co-emitted ions A and B in their coincidental mass spectrum. Using Equations S1 to S4, N_e is obtained as follows:

$$N_e = \frac{N_A \times N_B}{N_{A,B}} \tag{S4}$$

where N_A and N_B are the peak areas of ions A and B, respectively. The fractional coverage of specimens (K) on the surface area is given by:

$$K = \frac{N_e}{N_0} \times 100\% \tag{S5}$$

where N_0 is the total number of impacts. The effective number of impacts on a specimen (N_e) does not depend on the ionization probabilities and detection efficiencies of ions A and B. Thus, for surface objects that are larger than the emission volume; the fractional coverage can be calculated using the coincidental process[2,3].

The thiol-related ions SH^-, C_2HS^-, and AuS^- at m/z 33, 57 and 229, respectively, were chosen to calculate the surface coverage of coating on gold surfaces, K (%) using Equations from S3 to S5. Table S1 shows the surface coverage of linear-chain thiols used in this study.

Table S1. The surface coverage of coating layer on gold surfaces.

Sample	Surface coverage of thiol
**2-aminoethanethiol hydrochloride (–C_2NH_2)**	93 ± 5%
**16-mercaptohexadecanoic acid (–$C_{15}COOH$)**	92 ± 5%
**1-octanethiol (–C_7CH_3)**	95 ± 3%
**1-decanethiol (–C_9CH_3)**	92 ± 7%
**1-octadecanethiol (–$C_{17}CH_3$)**	93 ± 6%

2. Theory. For many colloidal systems (e.g., bacterial suspension) having repulsive interactions with a substrate, the particle adhesion (e.g., adsorption and deposition) process is first-order, which can be modelled as:

$$Particle + Substrate \longrightarrow Particle \bullet Substrate \tag{S6}$$

$$\frac{dN}{dt} = kn_b L(N) \tag{S7}$$

where N is the surface concentration of particles (number of particles/m^2), n_b is the bulk concentration of particles (number of particles/m^3), t is time (s), $L(N)=1-N/N_0$ is the Langmuir blocking function, N_0 is the saturation surface concentration, and k is the adsorption rate constant (m/s). The solution of the differential equation describes an exponential dependence between the surface concentration and time:

$$N = N_0(1 - e^{-\frac{k \times n_b}{N_0}t}) \tag{S8}$$

the adsorption constant, k, is strongly dependent upon the energetics between the adhering particles and the substrates, and also flow velocity and geometry when the suspending medium is not quiescent.

For colloidal adhesion processes with diffusion-dominant characteristics, which generally occurs when the total interaction between colloids and the substrate is attractive, the number of adhering colloids has a square root dependence on time, t[4,5]:

$$N = 2\left(\frac{D_\infty t}{\pi}\right)^{1/2} n_b \tag{S9}$$

where D_∞ is the constant diffusion coefficient in the suspension.

The interactions between bacteria and a substrate are often modeled by the DLVO theory, which assumes the superimposition of the van der Waals interactions and double-layer electrostatic interactions[6]. Depending upon the surface characteristics of the substrate and the concentration of electrolyte, the DLVO potential energy diagram can show: (i) a shallow secondary minimum at long distances, an energy barrier at intermediate distances, and a deep primary minimum at short distances; (ii) a shallow primary minimum at short distances, which is followed by a strong repulsive barrier at intermediate distances; or (iii) a deep primary minimum only at short distances. For some cases, additional corrections, which are important at short ranges, such as acid-base interactions[7], hydration interactions[8], and hydrophobic interactions[9] have been included in the total

energy considerations. However, the kinetic processes are generally governed by the energy barrier occurring at intermediate distances, which is weakly influenced by short-range interactions. As such, it is not unreasonable to neglect these short-range interactions while modeling the kinetics of initial stages of bacterial adhesion.

For cases where the electrostatic interactions are short-ranged in comparison to the size of bacterium, and electrolytes are monovalent, the double-layer energy between a bacterium and flat substrate with different charge densities can be expressed as follows[10,11]:

$$E_{DL} = 64\pi\varepsilon_0\varepsilon\left(\frac{k_BT}{e}\right)^2 R\left[\tanh\left(\frac{e\psi_1^0}{4k_BT}\right)\tanh\left(\frac{e\psi_2^0}{4k_BT}\right)\right]e^{-\kappa h} \quad \text{(S10)}$$

where R is the radius of bacterium, $1/\kappa$ is the Debye length (m), and h is the separation between the bacterium and surface.

For the geometry of a spherical bacterium near a flat substrate, the non-retarded van der Waals interactions can be described in terms of the Hamaker constant, A_H[12,13]:

$$E_{vdw} = -\frac{A_H}{6}\left[\frac{R}{h} + \frac{R}{2R+h} + ln\left(\frac{h}{2R+h}\right)\right] \quad \text{(S11)}$$

where A_H is the Hamaker constant, which typically ranges between 10^{-21} and 10^{-20} J for organic molecules in water[14,15]. The non-retarded Hamaker constant can be predicted either by the Lifshitz theory using the dielectric constants and refractive indexes of the materials and dispersing media[16] or from the measured surface tension of bacteria and the minimum equilibrium distance between two molecules, which is 0.165 nm[17].

Bacterial adhesion process involves not only substrate-bacteria interactions but also bacteria-bacteria interactions. DLVO interactions between a bacterium and a bacterium can be obtained by the multiplying DLVO interactions between a planar substrate and a bacterium with the Derjaguin factor[18]. However, another critical issue that must be considered here is bacterial deformations due to the domination of attractive van der Waals interactions over repulsive double-layer forces at short distances[19]. In this case, the non-retarded van der Waals interaction energy between a wall and a truncated sphere with a deformation (indentation depth), δ can be expressed as[20,21]:

$$E_{vdw\_def} = -\frac{A_HR}{6}\left(\frac{1}{x} + \frac{\delta}{x^2}\right) \quad \text{(S12)}$$

where x is the distance between the most deformed point of the truncated sphere and the wall. In addition, it is essential to consider elastic energy that must be spent to change in the shape of the

bacteria, which is roughly equal to[22]:

$$E_{elas} = \frac{8}{15} E^{*} \sqrt{R} \delta^{\frac{5}{2}} \quad \text{(S13)}$$

where $E*$ is the reduced Young's modulus and R the particle radius. Our high-resolution images revealed that the mean contact diameter due to the bacterial adhesion is 232.6 ± 25.4 nm for *S. aureus* (Fig. S5), corresponding to 18.5 ± 4.0 nm indentation depth via the Cord theorem. This value is comparable with the deformation of *S. aureus* on glass measured by Busscher and co-workers[22]. The reduced elastic modulus of *S. aureus* is ~47 kPa[22].

Overall, our modified DLVO model for the energetics of bacterial approach to a substrate includes three terms: double-layer interactions, van der Waals interactions, and elastic deformation energy. Since when deformations start to occur, the distance between bacteria and surfaces is small; double-layer interactions are much weaker than van der Waals interactions. Hence, the standard (non-deformed) electrostatic double-layer interactions can be used in the calculation of the total energy:

$$E_{tot} = E_{vdw\_def} + E_{DL} + E_{elas} \quad \text{(S14)}$$

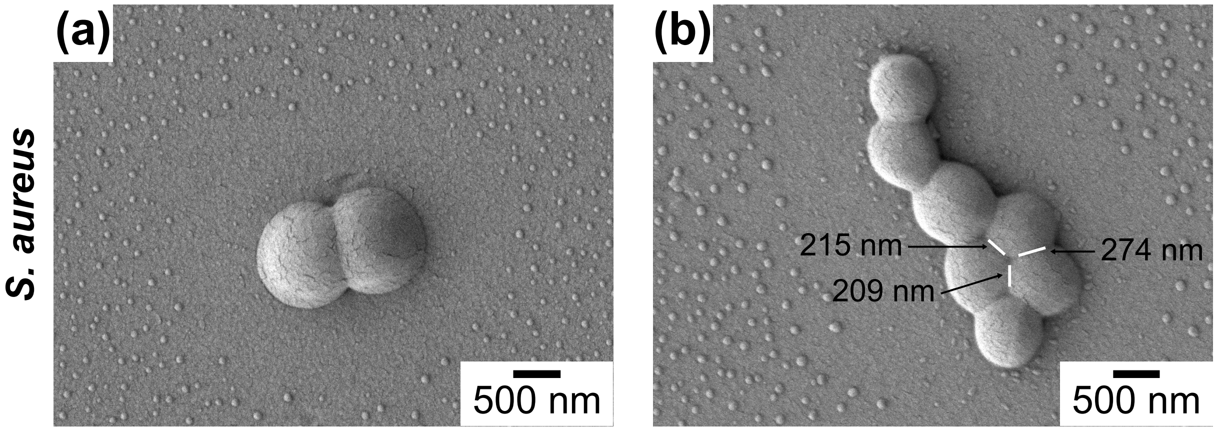

Figure S5. SEM micrographs of deformed *S. aureus* (a) during bacterial division and (b) upon adhesive contact with each other. The shape of contact curvature is used to distinguish these two phenomena

3. Estimation of interaction parameters. The effective electrolyte concentration of bacterial suspension was estimated from the electrical conductivity values, which were found to be 7.4 ± 0.4 × 10^{-4} S/m and 4.9 ± 0.3 × 10^{-4} S/m for *S. aureus* and *E. coli*, respectively. As the equivalent molar conductivity of most cations and anions lies in the range of 5 to 15 mS·m^2·mol^{-1}, we assumed that the suspension has an average molar conductivity of 10 mS·m^2·mol^{-1} and contains mostly monovalent ions[23]. This assumption yields an electrolyte (1:1) concentration of 3.7 × 10^{-5} M and 2.5 × 10^{-5} M for *S. aureus* and *E. coli*, respectively.

The Debye length (m) was calculated using the following expression[12]:

$$Debye\ Length = \kappa^{-1} = \left(\sum_i \frac{\rho_{\infty i} e^2 z_i^2}{\varepsilon_0 \varepsilon_r k_B T}\right)^{-1/2} \quad \text{(S15)}$$

where κ^{-1} is Debye length, ε_0 is the permeability of free space (8.85 × 10^{-12} F·m^{-1}), ε_r is the relative permeability of the buffer solution (78.4), k_B is the Boltzmann constant (1.38 × 10^{-23} J·K^{-1}), *T* is the temperature of the buffer (*K*), *e* is the elementary charge (1.6 × 10^{-19} C), ρ_∞ is the number density of ions of valency z for ith ion. The corresponding decay length was 5.0 × 10^{-8} m and 6.1×10^{-8} m for *S. aureus* and *E. coli* suspension, respectively.

For the interaction between material 1 (bacteria) and material 3 (substrate) across material 2 (water), the non-retarded Hamaker constant given by the Lifshitz theory is approximately[16]:

$$A_{H,123} = \frac{3}{4} k_B T \left(\frac{\epsilon_1 - \epsilon_2}{\epsilon_1 + \epsilon_2}\right)\left(\frac{\epsilon_3 - \epsilon_2}{\epsilon_3 + \epsilon_2}\right) + \frac{3hv_e}{8\sqrt{2}} \frac{(n_1^2 - n_2^2)(n_3^2 - n_2^2)}{\sqrt{(n_1^2 + n_2^2)}\sqrt{(n_3^2 + n_2^2)}(\sqrt{(n_1^2 + n_2^2)} + \sqrt{(n_3^2 + n_2^2)})} \quad \text{(S16)}$$

where the first term ($v = 0$) represents the permanent dipole and dipole-induced dipole interactions, and the second ($v > 0$) represents the London (dispersion) interactions. ε_i and n_i are the static dielectric constants and refractive indexes of the materials, respectively. v_e is the frequency of the lowest electron transition (~3 × 10^{15} s^{-1}). Using Equation S16, we estimated the Hamaker constant to in the range of 6.3 to 9.0 × 10^{-22} J depending on the substrate chemistry (Table S2).

Table S2. Dielectric and refractive index values used in estimating Hamaker constant for *S. aureus*/substrate and *S. aureus/S. aureus* systems[23–28].

**Material**		ε	n	**Hamaker constant ($\times 10^{-21}$ J)**
**Material 1 (Particle)**	**Bacteria**	18	1.380	·
**Material 2 (Medium)**	**Water**	78	1.330	·
**Material 3 (Coating)**	**$–C_2NH_2$**	2.1	1.435	3.3
**Material 3 (Coating)**	**$–C_{15}COOH$**	2.2	1.481	3.9
**Material 3 (Coating)**	**$–C_7CH_3$**	2.1	1.452	3.5
**Material 3 (Coating)**	**$–C_9CH_3$**	2.1	1.459	3.6
**Material 3 (Coating)**	**$–C_{17}CH_3$**	2.1	1.464	3.6
**Material 3 (Particle)**	**Bacteria**	18	1.380	1.9

kT (J) = 4.11×10^{-21}; v_e (s^{-1}) = 3.00×10^{15}; h (J·s) = 6.63×10^{-34}

4. Screening of antimicrobial activity. The antibiotic characteristics of thiol-coated substrates were investigated to determine whether bacterial adhesion and antimicrobial activity acted in concert or not for the model substrates selected. Bacterial suspensions of *S. aureus* and *E. coli* O157:H7 were grown in contact with thiol-coated substrates for 24 h at room temperature. Bacterial suspensions grown without substrates were treated as negative controls for each pathogen. The numbers of bacteria remaining in suspension following incubation was assessed by pour plate method. Each condition was replicated four times.

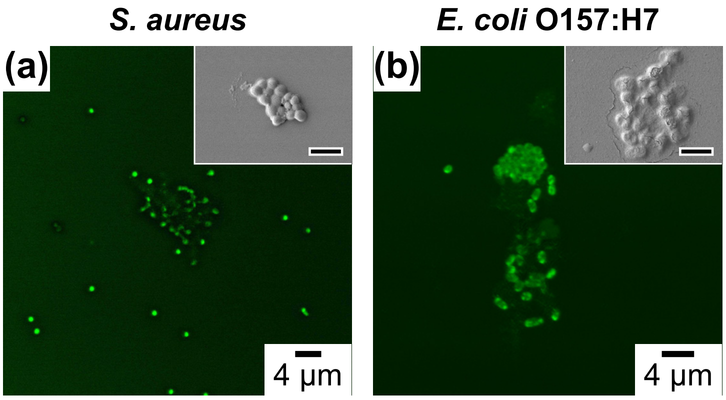

Figure S6. Fluorescence micrographs of the EPS (light green) and adhered cells (bright green). Calcofluor white dye was used to visualize the EPS produced by (a) *S. aureus* and (b) *E. coli* O157:H7. Upper insets: high magnification SEM micrographs of the EPS produced by *S. aureus* and *E. coli* O157:H7, respectively (scale bar, 3 μm).

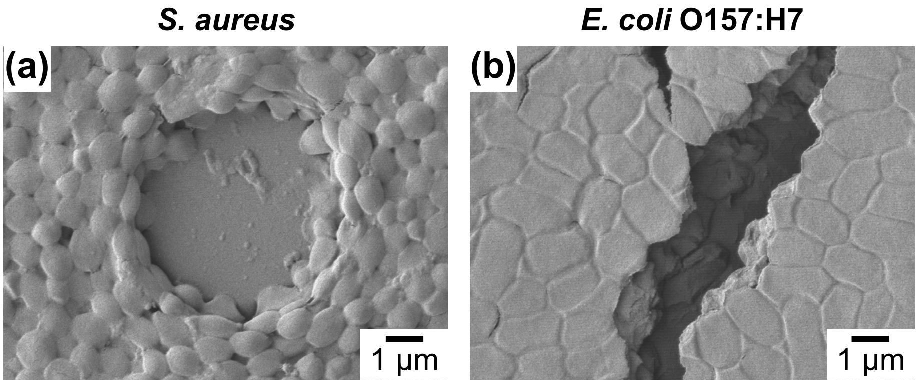

Figure S7. SEM micrographs of multilayered (a) *S. aureus* and (b) *E. coli* O157:H7 adhesion on bare gold surfaces by droplet inoculation method (no rinsing).

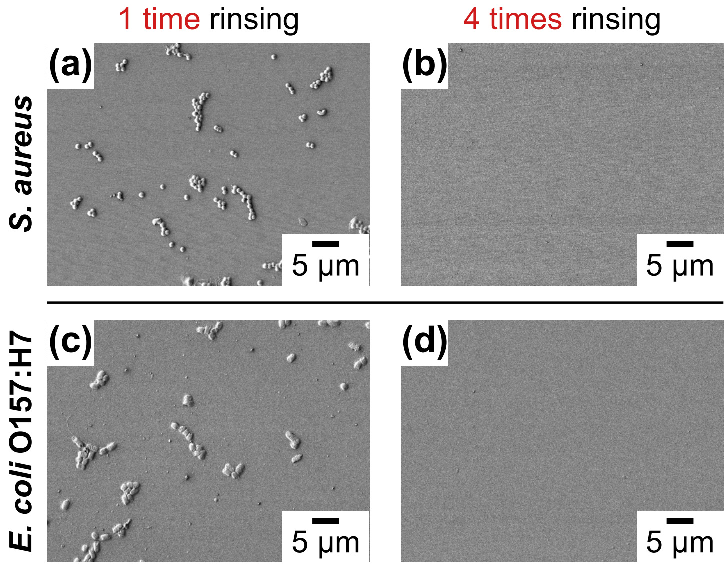

Figure S8. After specified incubation times, unbound (suspended cells) and loosely adsorbed bacteria on the surfaces were removed by rinsing 4 times with sterile distilled water. SEM micrographs of aliquots after (a),(c) 1 time rinsing and (b),(d) 4 times rinsing collected from the samples inoculated with *S. aureus* (top) and *E. coli* O157:H7 (bottom).

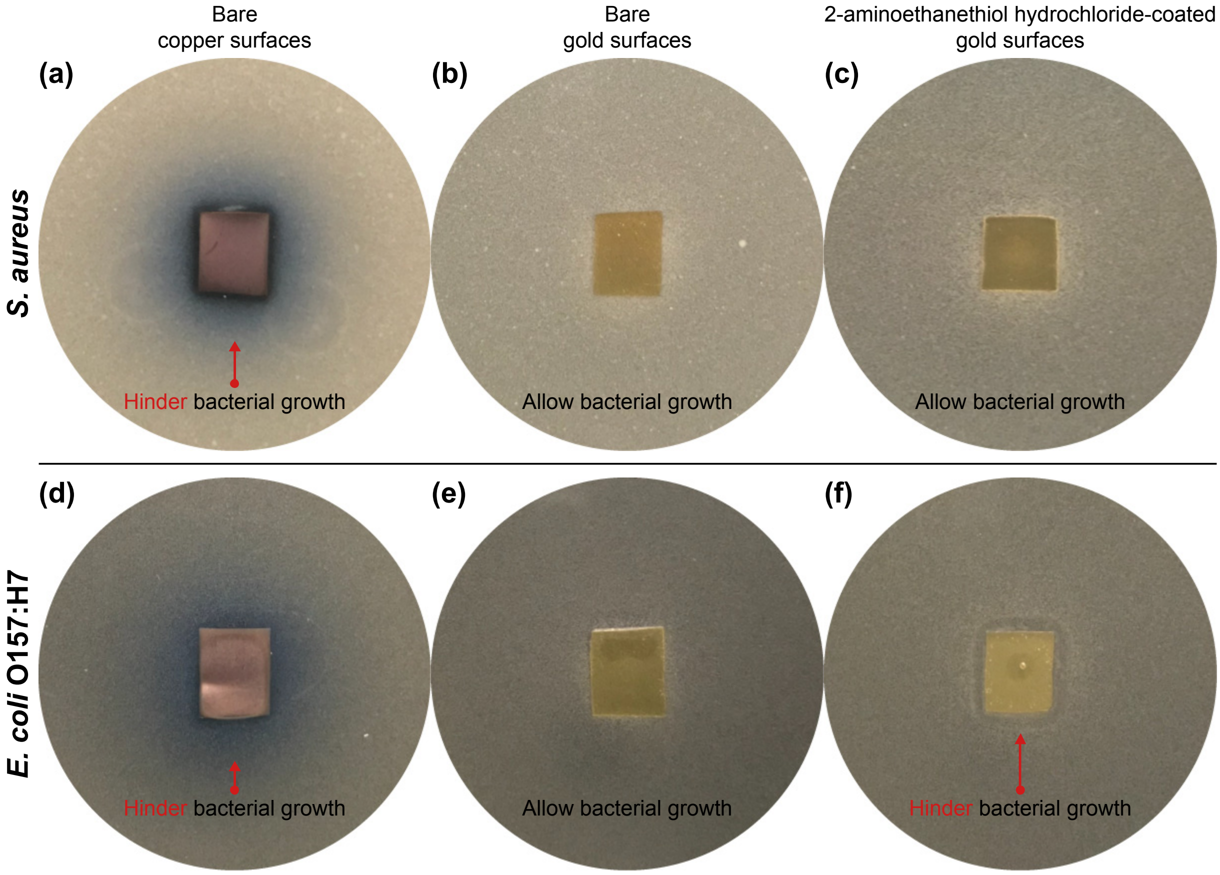

Figure S9. The disk-diffusion agar method tests of the bare copper, bare gold, and 2-aminoethanethiol hydrochloride-coated gold surfaces placed on the agar plate inoculated with *S. aureus* (top) and *E. coli* O157:H7 (bottom). Plate results indicate bacterial growth/no growth after 24 h on the (a),(d) bare copper, (b),(e) bare gold, and (c),(f) 2-aminoethanethiol hydrochloride-coated gold surfaces.

References

1. Yang, F. *et al.* Nanodomain analysis with cluster-SIMS: Application to the characterization of macromolecular brush architecture. *Surf. Interface Anal.* **47,** 1051–1055 (2015).

2. Chen, L.-J. *et al.* Characterization and quantification of nanoparticle–antibody conjugates on cells using C_{60} ToF SIMS in the event-by-event bombardment/detection mode. *Int. J. Mass Spectrom.* **303,** 97–102 (2011).

3. Rajagopalachary, S., Verkhoturov, S. V. & Schweikert, E. A. Characterization of individual ag nanoparticles and their chemical environment. *Anal. Chem.* **81,** 1089–1094 (2009).

4. Schwaab, M., Steffani, E., Barbosa-Coutinho, E. & Severo Júnior, J. B. Critical analysis of adsorption/diffusion modelling as a function of time square root. *Chem. Eng. Sci.* **173,** 179–186 (2017).

5. Miura, T. & Seki, K. Diffusion influenced adsorption kinetics. *J. Phys. Chem. B* **119,** 10954–10961 (2015).

6. Adamczyk, Z. Kinetics of diffusion-controlled adsorption of colloid particles and proteins. *J. Colloid Interface Sci.* **229,** 477–489 (2000).

7. Brant, J. A. & Childress, A. E. Assessing short-range membrane-colloid interactions using surface energetics. *J. Memb. Sci.* **203,** 257–273 (2002).

8. Israelachvili, J. & Wennerström, H. Role of hydration and water structure in biological and colloidal interactions. *Nature* **379,** 219–225 (1996).

9. Nel, A. E. *et al.* Understanding biophysicochemical interactions at the nano–bio interface. *Nat. Mater.* **8,** 543–557 (2009).

10. Prieve, D. C. & Ruckenstein, E. The surface potential of and double-layer interaction force between surfaces characterized by multiple ionizable groups. *J. Theor. Biol.* **56,** 205–228 (1976).

11. Chan, D., Healy, T. W. & White, L. R. Electrical double layer interactions under regulation by surface ionization equilibria–dissimilar amphoteric surfaces. *J. Chem. Soc. Faraday Trans. 1 Phys. Chem. Condens. Phases* **72,** 2844–2865 (1976).

12. Suzuki, A., Ho, N. F. H. & Higuchi, W. I. Predictions of the particle size distribution changes in emulsions and suspensions by digital computation. *J. Colloid Interface Sci.* **29,** 552–564 (1969).

13. Hamaker, H. C. The London—van der Waals attraction between spherical particles. *Physica* **4,** 1058–1072 (1937).

14. Baralia, G. G., Filiâtre, C., Nysten, B. & Jonas, A. M. Nanodecoding by dewetting. *Adv. Mater.* **19,** 4453–4459 (2007).

15. Bergström, L., Torbjörn Dahlfors, S. S., Arwin, H. & Ödberg, L. Spectroscopic ellipsometry characterisation and estimation of the Hamaker constant of cellulose. *Cellulose* **6,** 1–13 (1999).

16. Hough, D. B. & White, L. R. The calculation of Hamaker constants from Liftshitz theory with applications to wetting phenomena. *Adv. Colloid Interface Sci.* **14,** 3–41 (1980).

17. Israelachvili, J. N. in *Intermolecular and Surface Forces* 253–289 (Academic Press, 2011).

18. Adamczyk, Z. & Weroński, P. Application of the DLVO theory for particle deposition problems. *Adv. Colloid Interface Sci.* **83,** 137–226 (1999).

19. Chen, Y., Harapanahalli, A. K., Busscher, H. J., Norde, W. & van der Mei, H. C. Nanoscale cell wall deformation impacts long-range bacterial adhesion forces on surfaces. *Appl. Environ. Microbiol.* **80,** 637–643 (2014).

20. Forsyth, A. J. & Rhodes, M. J. A simple model incorporating the effects of deformation and asperities into the van der Waals force for macroscopic spherical solid particles. *J. Colloid Interface Sci.* **223,** 133–138 (2000).

21. Dahneke, B. The influence of flattening on the adhesion of particles. *J. Colloid Interface Sci.* **40,** 1–13 (1972).

22. Chen, Y., Norde, W., van der Mei, H. C. & Busscher, H. J. Bacterial cell surface deformation under external loading. *mBio* **3,** e00378-12 (2012).

23. Biagi, M. C. *et al.* Nanoscale electric permittivity of single bacterial cells at gigahertz frequencies by scanning microwave microscopy. *ACS Nano* **10,** 280–288 (2016).

24. Brayden, D. J., Rawlinson, L.-A. B., O'Gara, J. P. & Jones, D. S. Resistance of *Staphylococcus aureus* to the cationic antimicrobial agent poly(2-(dimethylamino ethyl)methacrylate) (pDMAEMA) is influenced by cell-surface charge and hydrophobicity. *J. Med. Microbiol.* **60,** 968–976 (2011).

25. Knoben, W., Brongersma, S. H. & Crego-Calama, M. Preparation and characterization of octadecanethiol self-assembled monolayers on indium arsenide (100). *J. Phys. Chem. C* **113,** 18331–18340 (2009).

26. Rusu, P. C. & Brocks, G. Surface dipoles and work functions of alkylthiolates and fluorinated alkylthiolates on Au(111). *J. Phys. Chem. B* **110,** 22628–22634 (2006).

27. van der Wal, A., Minor, M., Norde, W., Zehnder, A. J. B. & Lyklema, J. Conductivity and dielectric dispersion of Gram-positive bacterial cells. *J. Colloid Interface Sci.* **186,** 71–79 (1997).

28. Mathias, S. & Filho, E. D. C. The dipole moments of alkanethiols. *J. Phys. Chem.* **62,** 1427–1430 (1958).
